# Camman’s Stethescope

**Published:** 1855-10

**Authors:** 


### EDITORIAL.

*Camman's Stethescope.*—The inquiries of a correspondent remind us of a duty which should have been performed several months since. About one year since a neatly constructed Stethescope, invented by Dr. Camman of New York, and made by Tieman & Co. of the same city, was presented to the profession as an instrument much superior to those previously in use. We immediately procured one and have used it daily up to the present time.

It consists of two metallic and flexible tubes terminating in a funnel shaped ivory tube of large size, which is the part to be applied to the chest. The ends of the metallic tubes are terminated with ivory knobs of such size as to fit the opening of the external ear. The advantages which it possesses over most other Stethescopes, are, first the more full and perfect transmission of sound; and second the greater ease of adjustment. It being connected with both ears at the same time, more fully and plainly transmits the sounds to which you are listening, than any of those instruments which connect only with one ear of the operator.

The ease of adjustment arises from the fact, that the end to be applied to the chest is directly before the face of the operator and can be guided continually by the eye as well as the hand. Without entering further into details, we would say to our readers that the instrument is a very valuable one; and after using it long enough to become perfectly familiar with its qualities, we would not willingly part with it. It transmits the sounds, however, so differently from the old instrument, that it requires some practice to appreciate them correctly. This practice should be had first on the healthy chest. The instrument can be obtained of J. H. Reed & Co., Druggists of this city. Price $7,00.

